# Report upon Vaccination in the Royal Prussian Army

**Published:** 1855-07

**Authors:** 

**Affiliations:** Royal Brigade Physician in Aschersleben


### *Report upon Vaccination in the Royal Prussian Army.* By DR. SCHILLING, Royal Brigade Physician in Aschersleben.

(Translated for the Medical Examiner.)

Universally acknowledged and introduced, vaccination within fifty years has spread itself over the whole surface of the globe, and rendered complete by observation and experiment, has stripped the horrible plague, small pox, of its form of terror; and our only cause of regret is, that we are not in possession of similar protective weapons to oppose the other pestilential enemies of our race.

Those peculiarities of mind, closely allied to each other, that on the one hand prompt many to believe greedily in things the most marvellous and contradictory to their senses, as table moving, spirit rappings, clairvoyance, &c.; and on the other, cause some, like Schöpfer, in his proof of the static condition of the earth, to deny and treat as old delusions, facts that have been obtained by toilsome investigation and are recognized as established truths, and are among the characteristics of our age. Thus, not only are the beneficent results of this discovery now placed in question, but vaccination is even arraigned before the tribunal charged with the greatest crimes, namely, of having for fifty years been engaged in poisoning the population of the world.

It is not, of course, my intention to answer these charges, nor to undertake to prove that cholera, typhus, scrofula, scarlatina, croup, hydrocephalus, tuberculosis and its kindred evils, by whatever name they may be called, have not entered upon the stage of human misery, in consequence of the repeated inoculation of the variolous poison; that would be a work of supererogation. Besides, wherever justice rules, the custom is well established, that the prosecutor cannot merely accuse and leave to the defendant the task of proving his innocence, but the accuser must furnish the proof of his assertion. My purpose is simply to furnish a statistical contribution to the vaccination question that has of late repeatedly become a subject of debate. It is my object to prove the protective power of re-vaccination by the statistics of a military district in my neighborhood, embracing a period of 20 years, and of 859,880 re-vaccinations. Whether we view re-vaccination as merely rendering previous vaccination complete, or as proving the entire absence of the same, is a matter of indifference.

By the Supreme Cabinet order, dated June 30th, 1826, it was ordered that all persons in the military service should be vaccinated, who had not previously been vaccinated, or who did not bear upon themselves perceptible marks therefrom. Eight years later, doubtless from the observations made regarding the loss of the protective power from the lapse of years, the Supreme Cabinet orders of June 16th, 1834, commanded the re-vaccination of all troops, without distinction, upon their enlistment, whether they bore marks of vaccination or not. Those persons only were excepted who already showed the unmistakeable scars of small pox. In section 4 of the circular of the Minister of War, it was ordered that the lymph should be obtained from youthful subjects vaccinated for the first time. By a circular from the chief of the military medical department, dated May 12th, 1837, it was made known that according to the results of numerous experiments made with lymph obtained from adult cases of genuine cow pox, re-vaccination quite as often gave rise to beautiful and regular pustules, running their usual course, possessing true protective powers, and with even a more lively reaction than usual.

In accordance with the above regulations, for a long series of years, all the troops enlisted were re-vaccinated by the military physicians, as soon after their assumption of the uniform as possible, with the single exception of those bearing positive signs of a previous attack of small pox. From the complete report of the physicians-in-chief of the different portions of the army, the following tables were constructed under the title of "The results of re-vaccination in the Army." Owing to the number of physicians (at least 300, including assistant physicians,) who took part in the inoculating and in watching the results of the same, all discrepancies and errors were removed, regarding the existence of the scars of prior vaccination, and of the regularity or irregularity of the course of the new vaccination. I now give the results of re-vaccination in the army from the years 1833 to 1852, both inclusive.

### *Results of Revaccination in the Army.*

| 1 | 2 | 3 | | | 4 | | | 5 | | 6 | | | | 7 | | | 8 |
|---|---|---|---|---|---|---|---|---|---|---|---|---|---|---|---|---|---|
| | | Scars showing previous vaccination. | | | Of the new vaccination, the course was as follows: | | | Of those without result, were repeated: | | Number of genuine pustules obtained by the vaccination. | | | | Statement of the number of successful cases of revaccination that were attended with variolous diseases. | | | Out of 100, true pustules appeared in |
| | | a. | b. | c. | a. | b. | c. | a. | b. | a. | b. | c. | d. | a. | b. | c. | |
| Year. | Number of Vaccinated. | Decided. | Uncertain. | None. | Regular | Irregular. | No result. | With success. | Without success. | 1–5 | 6—10 | 11—20 | 21—30 | Varicella. | Varioloid. | Genuine variola. | |
| 1833 | 48,478 | 37,286 | 7,641 | 3,551 | 15,269 | 12,203 | 21,006 | 784 | 3,377 | 6,586 | 4,854 | 3,217 | 612 | 54 | 50 | 20 | over 33 |
| 1834 | 44,454 | 33,634 | 7,134 | 3,686 | 16,679 | 12,287 | 15,488 | 866 | 3,664 | 6,763 | 5,028 | 4,088 | 800 | 46 | 31 | 2 | " 39 |
| 1835 | 39,192 | 30,242 | 6,233 | 2,717 | 15,315 | 10,135 | 13,742 | 1,465 | 7,946 | 6,298 | 4,990 | 3,417 | 610 | 21 | 14 | 1 | almost 43 |
| 1836 | 42,124 | 32,635 | 6,645 | 2,844 | 18,136 | 9,940 | 14,048 | 1,569 | 8,205 | 7,311 | 5,647 | 4,418 | 760 | 14 | 8 | — | over 46 |
| 1837 | 47,258 | 37,299 | 6,903 | 3,056 | 21,308 | 10,557 | 15,393 | 2,243 | 9,771 | 9,174 | 6,414 | 4,767 | 953 | 14 | 7 | — | " 40 |
| 1838 | 42,041 | 33,819 | 5,645 | 2,577 | 19,117 | 8,672 | 14,252 | 2,306 | 10,424 | 8,787 | 5,581 | 4,056 | 693 | 19 | 10 | 2 | almost 51 |
| 1839 | 41,481 | 33,225 | 5,889 | 2,367 | 19,249 | 8,534 | 13,698 | 2,105 | 7,886 | 8,762 | 5,650 | 4,095 | 742 | 18 | 7 | — | over 51 |
| 1840 | 43,522 | 34,573 | 6,177 | 2,772 | 20,952 | 8,820 | 13,750 | 2,831 | 8,958 | 10,021 | 5,875 | 4,171 | 885 | 7 | 2 | 1 | " 54 |
| 1841 | 44,941 | 36,182 | 6,192 | 2,567 | 23,383 | 8,035 | 13,523 | 2,254 | 9,468 | 11,174 | 6,516 | 4,838 | 855 | 1 | 8 | — | " 57 |
| 1842 | 42,582 | 33,185 | 6,751 | 2,646 | 21,865 | 8,056 | 12,661 | 2,029 | 9,536 | 10,697 | 6,223 | 4,200 | 745 | 9 | 4 | — | " 58 |
| 1843 | 42,998 | 34,390 | 6,258 | 2,350 | 22,062 | 8,613 | 12,323 | 2,439 | 9,671 | 10,568 | 6,426 | 4,392 | 676 | 11 | 8 | 4 | almost 57 |
| 1844 | 40,661 | 32,779 | 5,463 | 2,419 | 21,038 | 7,945 | 11,678 | 2,278 | 9,712 | 11,571 | 6,619 | 4,417 | 709 | 5 | 8 | 1 | over 57 |
| 1845 | 42,671 | 33,813 | 6,041 | 2,817 | 22,214 | 8,764 | 11,693 | 2,749 | 9,974 | 12,208 | 6,944 | 4,917 | 894 | 2 | 3 | — | " 58 |
| 1846 | 44,012 | 34,708 | 6,453 | 2,851 | 24,138 | 7,991 | 11,883 | 2,534 | 9,855 | 13,213 | 7,510 | 5,081 | 868 | 4 | — | — | " 60 |
| 1847 | 43,596 | 34,264 | 6,405 | 2,927 | 25,544 | 7,425 | 10,627 | 2,718 | 8,952 | 13,295 | 8,164 | 5,767 | 1,036 | — | 1 | — | almost 65 |
| 1848 | 28,859 | 22,386 | 4,211 | 2,262 | 16,882 | 4,404 | 7,573 | 1,579 | 5,786 | 9,161 | 5,134 | 3,513 | 653 | 6 | 1 | — | " 64 |
| 1849 | 51,637 | 39,116 | 8,706 | 3,815 | 30,457 | 8,467 | 12,713 | 2,862 | 9,194 | 16,018 | 9,621 | 6,617 | 1,063 | 5 | 9 | 1 | 64 |
| 1850 | 44,539 | 33,466 | 7,053 | 4,040 | 25,030 | 7,509 | 12,000 | 2,355 | 8,766 | 12,461 | 7,964 | 6,092 | 868 | 10 | 22 | — | 61 |
| 1851 | 57,059 | 42,203 | 9,935 | 4,921 | 33,444 | 9,857 | 13,758 | 3,338 | 10,354 | 16,203 | 11,296 | 8,196 | 1,087 | 20 | 41 | — | 64 |
| 1852 | 27,775 | 21,195 | 4,242 | 2,338 | 17,782 | 3,920 | 6,073 | 1,468 | 4,639 | 8,413 | 5,967 | 4,304 | 564 | 5 | 7 | — | 69 |

This summary shows the total number of vaccinations during 20 years to have been 859,880) (1st column.) Among these, 429,864 yielded successful cases, regular in their course, (column 4, a.) Those remaining without result, but re-vaccinated afterwards, successfully, amounted to 43,770, (column 5, a.) So that in all, 473,634 cases of genuine and regular vaccination were produced.

Of those successfully re-vaccinated during 20 years, 545 were attacked with (column 7)

| | | |
|---|---|---|
| Varicella, | 271 | Column 7, a. |
| Varioloid, | 241 | " 7, b. |
| Variola, | 33 | " 7, c. |

The year 1833 is strikingly remarkable as being by far the most unfavorable in its protective results. It would have been better to have omitted the first year, and only cited the last 19 years, if I had only wished to produce the proofs of a statement, and not, at the same time, to make the utmost use of the whole of the materials at my command. But I must remember that this universal vaccination, without regard to whether it had been previously done or not, only commenced with the year 1834. A proper estimation of re-vaccination as it now exists in the army, can, in fact, therefore be obtained by taking into account the last 19 years only.

In these 19 years, the number vaccinated upon their entrance into the army was 811,402, (column 1.) The first vaccination was successful with 414,595, (column 4, a.) Those unsuccessful at first, but successfully re-vaccinated, were 42,986, (column 5, a.) The vaccination, therefore, took effect in a regular manner with 457,581. Of those successfully re-vaccinated during 19 years, 421 were attacked in 19 years

| | |
|---|---|
| With Varicella, | 217. |
| Varioloid, | 191. |
| Variola, | 13. |

Of these 457,581 soldiers successfully vaccinated during 19 years, 4 have died of variola.

This result is indeed astonishingly favorable, for variolous diseases still exist and appear in all times and all places in our State, notwithstanding that compulsory vaccination and the extinction of prejudice have diminished from year to year the number of those who have avoided vaccination. An immunity from the contagion of variolous diseases exists neither in age, sex, race nor constitution. The rate of mortality of the different epidemics, it is true, is very different, sometimes rising to 60 or 70 per cent., sometimes sinking to 15 per cent. of those attacked. The average mortality may be stated as about 30 per cent. The Prussian army, however, represents the male portion of the population of the State in the bloom and manly vigor of their age, dwelling in all parts of the country. They live distributed among the rest of the population, in part sharing the house with the citizen, therefore in the most favorable position for infection, and, in part, crowded together in barracks, therefore in a state best adapted to the further distribution of the disease. It has never failed to contribute its share to the increase of other epidemic diseases, when they have appeared ; for instance, to typhus and cholera. With reference to the liability to variolous diseases, I here give the not inconsiderable number of cases in the whole army, without regard as to the result of the vaccination. In the whole army there were attacked with variolous diseases,

| A. | B. | C. | Of the number revaccinated. | | |
|---|---|---|---|---|---|
| Year. | Attacked | Died. | D. With success. | E. Without success. | F. No result. |
| 1834 . . . . . . . . . . | 619 | 38 | 2 | 6 | 30 |
| 1835 . . . . . . . . . . | 259 | 5 | — | 2 | 3 |
| 1836 . . . . . . . . . . | 130 | 9 | ? | ? | ? |
| 1837 . . . . . . . . . . | 94 | 3 | — | — | 3 |
| 1838 . . . . . . . . . . | 111 | 7 | — | 2 | 5 |
| 1839 . . . . . . . . . . | 89 | 2 | — | — | 2 |
| 1840 . . . . . . . . . . | 74 | 2 | — | — | 2 |
| 1841 . . . . . . . . . . | 59 | 3 | — | 1 | 2 |
| 1842 . . . . . . . . . . | 99 | 2 | — | 1 | 1 |
| 1843 . . . . . . . . . . | 167 | 3 | 1 | 2 | — |
| 1844 . . . . . . . . . . | 69 | 3 | 1 | 2 | — |
| 1845 . . . . . . . . . . | 30 | 1 | — | 1 ? | — |
| 1846 . . . . . . . . . . | 30 | 1 | — | 1 | — |
| 1847 . . . . . . . . . . | 5 | — | — | — | — |
| 1848 . . . . . . . . . . | 22 | 1 | — | 1 | — |
| 1849 . . . . . . . . . . | 62 | 1 | — | — | 1 |
| 1850 . . . . . . . . . . | 176 | 1 | — | — | 1 |
| 1851 . . . . . . . . . . | 246 | 3 | — | 2 | 1 |
| 1852 . . . . . . . . . . | 87 | 1 | — | 1 | — |
| Total . . . . | 2428 | 86 | 4 ? | 22 ? | 51 ? |
| Or, if we distribute the 9 who died in 1836 between the columns E and F | | | | | |
| The total would be . . . . | 2428 | 86 | 4 | 25 | 57 |

NOTE.—Of these 86 that have died, at least 5 had had natural small pox, and on that account were not vaccinated, (in the early years no statement thereof was made,) while during the same time only 4 died who were successfully re-vaccinated.

There can be no other means of explaining this small mortality from variolous diseases in the army except by re-vaccination, whose action, as the figures in the above columns show, is expressed in a two-fold manner. 1st, that it reduces to a minimum the number of those attacked by the disease, so far as the re-vaccination was successful; and 2d, that it deprives the disease in those who were attacked of its prognosis, otherwise so unfavorable.

These results confirm what has already been shown by experience, viz., that vaccinia exerts its protective effect especially towards variola, and in a much less degree towards varioloid and varicella. It must be borne in mind, however, that whilst the mortality in variola is 30 per cent. and in varioloid 10 to 15 per cent., that during 19 years, out of 2428 cases that occurred in spite of successful re-vaccination, the mortality was only 86; showing, at least, a most decided mitigating influence produced by vaccination upon the course of the varieties of variolous diseases.

One of the most important questions in the thema of vaccination has been at all times, and more especially at present, the duration of the productive power of vaccinia. Periods, the most variable and arbitrarily assumed, have been given as the maximum of its duration. Upon this point, however, the tables thus obtained can give no positive conclusions, since by far the larger part of our army (that is with only the exception of the "*chargirten*,") is completely renewed in three years, at furthest. Positive results can, therefore, only be obtained from these figures with regard to the continuation of the protective power for the space of three years.

The results given in the last column are, however, most striking. It shows an almost uninterruptedly progressive increase from year to year of the protective vaccinia obtained by vaccination, so that the per centage of successful vaccination, out of the total number of vaccinations during 20 years, has increased from 33 per cent. to 69 per cent., or in 19 years, from 39 to

69 per cent., an annual increase of from 1 4-5th to 1½ per cent. respectively.

In my opinion there can be but two methods of explaining this phenomenon ; either the method of vaccination must have been improved, or the sensitiveness of the present generation to the vaccine virus must have increased. To the first explanation, the following thoughts naturally oppose themselves, viz., that the act of the inoculation itself and the observation of its results, are so extremely simple, that although *two* physicians may form widely different deductions therefrom, yet it is impossible for any *three hundred* physicians to obtain results essentially different from those obtained by any other three hundred. I know further from my own personal observation and practice in our army for the last 10 years, that the mode of vaccination has been for that period precisely the same as it is at this day. The rate of increase, however, in the number of successful results, was the same in those 10 years previous with which I had no personal acquintance, as it was in the time that *has* come under my actual notice; I do not think I risk anything in stating, therefore, that the method of vaccination did not exert any influence upon the results obtained.

This leaves us only the supposition of an increased susceptibility in this generation to the vaccine influence.

Increased susceptibility in re-vaccination will appear to many persons as identical with increased liability to the infection of small pox, and may lead to erroneous conclusions, for it is often considered as certainly established that a successful vaccination was a proof of previous susceptibility to the influence of the variola infection, while, on the contrary, those particularly that have been carefully repeated without success, are looked upon as evidences of a want of susceptibility of the individual to the small pox contagion.

If the above views are correct, the protective results of vaccination in the *whole* army, taken together, would be the same as it was in those where successful results appeared, that is out of the whole number who were vaccinated on entering the army, (811,402 individuals,) the proportionate number, viz., 7⅙ persons, would have died, 4 only having died out of 457,581, successfully re-vaccinated.

The columns D. and E. of the tables show that out of the total number of persons who were vaccinated, 29 died, 811,402—457,581=353,821 that were vaccinated without success, out of whom 24 died of variolous disease. A positive proof that *successful* re-vaccination affords the only actual, and, for a certain time, *absolute* protection.—*Vierteljahrsschrift für gericht. und öffent. Medecin. Oct.* 1854.

